# Improving Zn Anode Cyclability in Alkaline Electrolytes with Electropolymerized Anion-Selective Film

**DOI:** 10.1021/acsaem.5c02209

**Published:** 2025-11-18

**Authors:** Elisa Emanuele, Claudio Mele, Benedetto Bozzini

**Affiliations:** 1 Department of Energy, 18981 Politecnico di Milano, via Lambuschini 4, Milano 20156, Italy; 2 Department of Innovation Engineering, 201812 University of Salento, Via Monteroni, Lecce 73100, Italy

**Keywords:** Zn secondary batteries, Zn−Ni, electropolymerization, anionic membrane, Zn Anode, RZAB

## Abstract

Zinc-based batteries are considered a promising alternative for sustainable energy storage due to their low cost and environmental benefits. However, challenges such as dendrite formation, passivation, and zincate migration hinder their performance and long-term stability. In this study, we address these issues by developing anion-selective poly­(vinylbenzyltrimethylammonium chloride) membranes via cathodic electropolymerization to modify zinc anode. These films were designed to confine zincate ions and reduce passivation and shape change during cycling while preventing self-discharge and avoiding the contact between metallic Zn and active water. Under extremely harsh testing conditions (closed cell, small amount of electrolyte), the Zn anode is modified with electropolymerized polymer and shows significantly improved performance compared to bare Zn foil. The highly rechargeable Zn anode reported is an important step toward practical Zn secondary aqueous batteries. This work proposes an improved, water-based strategy for zinc anode modification with electropolymerized anion-exchange films that improves the performance in alkaline electrolytes, as demonstrated by fundamental electrochemical tests and cycling of Zn–Ni batteries.

## Graphical abstract

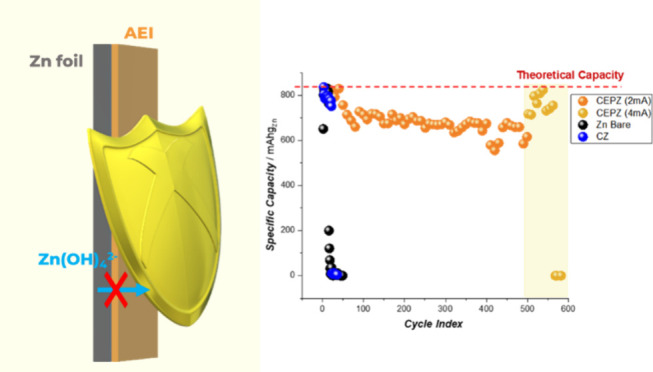

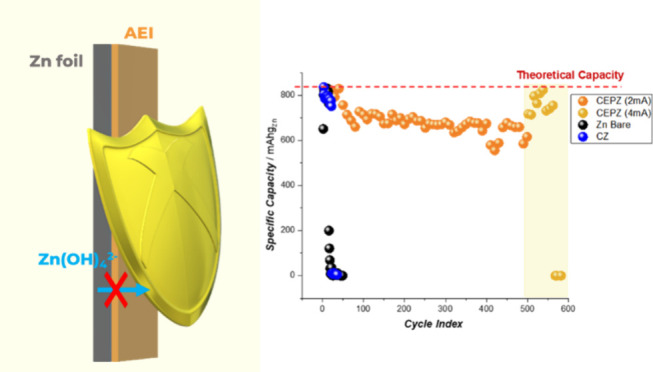

## 1 Introduction

The increasing need for efficient and sustainable energy storage solutions has driven significant research into a wide range of battery technologies. Rechargeable zinc batteries have gained attention as promising alternatives thanks to their availability, low cost, and eco-friendly nature. Bulk metallic Zn foil is the standard anode material that can be coupled with different types of cathodes, leading to the development of different batteries chemistries and geometries. The key Zn-based alkaline batteries, developed over the last few decades, are Zn/NiOOH, Zn/MnO_2_, Zn/Ag_2_O, and Zn/O_2_.^1–3^ However, the commercialization of these secondary batteries has been hindered by cycling stability issues arising mainly,^4–6^ though not exclusively,^7,8^ from the anode.

The development of a reliable and long-lasting zinc anode faces critical challenges, mainly the hydrogen evolution reaction (HER) and dendrite growth during charge and passivation during discharge. Alkaline Zn-batteries typically employ a 4–10 M KOH aqueous solutions, which have a narrow electrochemical stability window (ESW) of approximately 1.5 V, limited by HER and OER, the former being more common in practical conditions and leading to capacity loss and pressure buildup, both impacting durability.

Additionally, during discharge, metallic Zn undergoes electrochemical oxidation to form zincate ions [Zn­(OH)_4_]^2–^. Concentration gradients of these ions, combined with surface and electric field heterogeneties, can trigger shape changes. Furthermore, zincate can form insoluble zinc oxide (ZnO) passivation layers of micrometric thickness on the anode as a result of synergistic electrokinetic, mass transport, and precipitation processes. Once formed, this passivation layer inhibits further zinc oxidation by restricting access to the underlying zinc, ultimately impacting the efficiency, capacity, and cycling stability of the battery.^9^ Moreover, when considering the full battery, zincate migration can poison cathodes of specific chemistries, prototypically MnO_*x*_-based ones. In fact, zincates tend to react with soluble Mn^3^^+^ intermediates, forming an electrochemically inactive and insulating amorphous phase of ZnMn_2_O_4_ (hetaerolite).^2,8^ Common separators, such as commercial polyolefin, nonwoven, and glass fiber materials, are unable to impede the transport of zincate anions formed during discharge from the anode to the cathode. Moreover, even in cases where zincate does not react with the cathodic material, its crossover remains problematic, leading to the loss of active material and, as hinted at above, irregular deposition that contributes to dendrite growth. A confinement barrier that can keep zincate near the electrode surface is a possible strategy to mitigate these challenges and enable better cycling stability. In particular, anion-exchange ionomer (AEI) membranes, placed between the Zn anode and the separator, are in principle capable of confining larger zincate ions and allowing smaller hydroxide ions to permeate. Commercial anion-exchange ionomers, including Tokuyama AS4 and Fumasep FAA3, have been successfully applied in Zn–Ni and Zn–air systems.^2^ Ionomers are used either as a coating^10–12^ of the anode or as a binder in slurry-based electrodes.^13^ Shroder et al. investigated the AEI coating approach, starting with fundamental potentiodynamic studies on Zn wire^10^ and later extending the study to practical applications using a Zn-sponge^11^ and ZnO-coated carbon cloth electrodes.^12^

Shroder et al. obtained a homogeneous AEI coating using a drop-casting method, dipping the Zn electrodes into a volume of approximately 5 mL of AEI solution for 1 s before withdrawing them within the same time frame.

Electropolymerization (EP) is a suitable alternative method for fabricating anion-conducting polymer films.^15–17^ In this process, a redox reaction involving an organic molecule (the monomer) is used to directly form a polymeric film, typically well-adhered to the electrode. Electrochemical methods are intrinsically green, considering that the process is rapid and conducted under mild conditions, eliminating the need for additional steps for extraction and purification, which enhances the feasibility and reduces costs. Moreover, EP holds a distinct advantage over the method of evaporating a polymer solution onto a metal surface, primarily due to its superior control over thickness and structure, of course, if the EP mechanism at stake is appropriately understood and its capability to coat metallic components of various shapes. EP was initially and primarily used for the electrochemical synthesis of electrically conductive polymers like bithiophene,^14,18^ as these polymers possess electron conductivity, allowing the polymer film to grow continuously on the electrode with minimal self-limiting issues. Examples of electropolymerization of anion-selective polymer films on Zn metal are reported in the literature with the purpose of improving corrosion resistance.^19,20^

In this work, we performed cathodic EP to synthesize anion-conducting polymer films starting from the monomer vinylbenzyltrimethylammonium chloride (VBTMA). This type of molecule was chosen because the vinyl double bond present can be either reduced or oxidized to form a radical anion or a radical cation. Thus, VBTMA can be electropolymerized both cathodically and anodically. Such an aspect is fundamental because electrodeposition on Zn can only be performed cathodically due to the corrosion of the metal at −1.2 V vs Ag/AgCl in neutral pH. Since EP conditions are crucial for the formation of the polymeric film, the choice of electrochemical parameters, such as solvents, supporting electrolytes, monomer concentration, and the electrochemical method applied (potentiostatic or potentiodynamic conditions), is essential. For this reason, we first fully characterized the electrochemical behavior of VBTMA at an inert electrode (glassy carbon, GC) and we then found the best conditions for electrodeposition onto Zn foil electrodes. Recent work by Di Vona et al.^21^ has demonstrated for the first time the electropolymerization of VBTMA directly onto Zn anodes for rechargeable Zn–air batteries, employing cyclic voltammetry in a DMSO- based electrolyte for the electropolymerization process.

In the present work, we extend this approach by developing a water/ethanol-based electropolymerization process that yields more stable films and by demonstrating their effectiveness in the harsher (closed cell, small amount of electrolyte) alkaline environment of Zn–Ni full cells. In addition, we uncover a synergistic effect between the electropolymerized membrane and a hot-pressed microporous separator, leading to stability improvements beyond what either component can achieve alone.

This study encompasses (i) electrochemical characterization of the VBTMA monomer; (ii) electrodeposition of anion-selective membranes, (iii) electrochemical and functional characterization of the membranes, and (iv) the implementation of an EP membrane on Zn to tackle Zn anode stability challenges. Specifically, we employed (i) Raman spectroscopy to assess the chemical evolution of the membranes, (ii) cyclic voltammetry to characterize the electrochemical activity of the monomer and its polymerization, and (iii) electrochemical impedance spectrometry to investigate the growth and transport properties of the electrodeposited polymer films. Additionally, we performed long-term galvanostatic charge–discharge cycling tests to evaluate the stability and cycling performance of the Zn anodes coated with anion-exchange polymer coatings in full-cell configurations. These combined techniques provided comprehensive insight into the formation, electrochemical behavior, and cycling performance of the electropolymerized coating, highlighting its potential in improving the efficiency and longevity of Zn-based electrochemical energy storage systems.

## 2 Materials and Methods

### 2.1 Electrode Preparation

AEI membranes were directly formed on the Zn foil electrode, as described in Section 2.2.2(ii)*.* As-coated Zn foils were directly used as anodes, denominated EP-polymer-coated Zn (**EPZ**). A second series of modified Zn electrodes, denominated “dip-coated EPZ” (**DEPZ**), was prepared by dip-coating EPZ electrodes with PiperION polymer (PiperION Anion Exchange Dispersion, 5 wt %, fuel cell store). Dip coating was carried out by immersion into the as-received PiperION solution for 30 s and then drying in a vacuum oven at 70 °C for 1 h. Moreover, electrodes with a dip-coated membrane were also employed, which will be denominated ″dip-coated Zn” (**DPZ).**

A third series of anodes, denominated “Celgar-wrapped EPZ” (**CEPZ**), were prepared by wrapping EPZ electrodes with three foils of Celgard (CELGARD Polypore company 3501). The CEPZ was then hot-pressed at 60 °C using a laboratory hydraulic press (YLJ-HP88 V-350) for 3 min at 70 kg/cm^2^ and 1 min at 210 kg/cm^2^ to ensure good adhesion to the Zn foil. This two-step hot-pressing protocol was developed heuristically: The initial low-pressure step promotes uniform interfacial contact, while the subsequent high-pressure step ensures strong adhesion and mechanical stability. Trials performed using only low or high pressure did not yield a comparable adhesion quality.

For the sake of comparison, an anode with bare Zn foil wrapped with Celgard, applied with the same procedure explained above, was also prepared that will be called (**CZ**).

### 2.2 Electrochemical Measurements

The Zn substrates were polished with SiC paper and washed ultrasonically for 5 min with acetone and then water before each measurement.

#### 2.2.1 Monomer Electrochemical Characterization

The redox properties of VBTMA were studied in aprotic dimethyl sulfoxide (DMSO) (Merck, ACS reagent, ≥99.9%) and aqueous H_2_O:EtOH (9:1 volume ratio) solvents containing 1 mM VBTMA with 50 mM LiClO_4_ (Merck, ACS reagent, ≥95.0%) or NH_4_Cl (Merck, ACS reagent, ≥99.5%), respectively, as supporting electrolytes. The solutions were deaerated by N_2_ bubbling followed by coverage with an N_2_ blanket. Electrochemical measurements were carried out in a (standard AMEL) electrochemical cell containing about 10 mL of solution. Cyclic voltammetry (CV) and chromatoamperometry (CA) measurements were carried out in stagnant electrolyte at glassy carbon (GC), Pt, and Zn electrodes. The electrochemical measurements were carried out with a VMP-300 BioLogic potentiostat. The CVs were recorded at the scan rates in the range 0.05–1.0 V s^–1^ with terminal voltages selected as discussed, which were relevant for the characterization of each redox step. The recorded potentials are referenced to the Fc^+^|Fc (ferrocenium|ferrocene) redox couple added as external standard in a blank solution at the end of each daily measure session.

#### 2.2.2 Electropolymerization

Electrodepositions of VBTMA films on the GC, Pt, and Zn electrodes were performed by two protocols.

(i) Potentiodynamic protocol with 1 mM VBTMA in 250 mM LiClO_4_ DMSO. Twenty consecutive reductive potential cycles at 20 mV s^–1^ were performed around the first cathodic peak involving the reduction of the vinyl group, as specified in Section 3.2.1. After electrodeposition of the AEI film, the electrode was washed with acetone and DI water.

(ii) Potentiostatic protocol with 0.25 M VBTMA in 50 mM NH_4_Cl H_2_O:EtOH. Potentiostatic experiments were carried out under magnetic stirring in the range of −1.37 to −1.6 V vs Ag/AgCl at 50 °C for 15 min: More details on potential choice are provided in Section 3.2.2.

Protocol (ii) employing a potentiostatic deposition at −1.47 V vs Ag/AgCl was selected for all subsequent electrochemical tests.

#### 2.2.3 Electrochemical Confinement Test

All measurements were conducted in the cell described in Section 2.2.1 in aqueous KOH (4 M), employing an aliquot of fresh electrolyte for each test. The WE were Zn electrodes prepared as detailed in Section 2.1, the RE was an Hg/HgO (AMEL 383/OHG/12, 0.1 M KOH), and the CE was a Pt wire. Confinement experiments consisted of running 50 CV cycles at 4 mV s^–1^ under stirring, scanning from open circuit potential (OCP) to −0.65 V vs Hg/HgO in the anodic sweep, followed by scanning to −1.8 V vs Hg/HgO in the cathodic sweep, and finally back to OCP.

#### 2.2.4 Galvanostatic Cycling with Potential Limitation (GCPL)

Cycling tests were carried out in split cells (EL-CELL, ECC-std) using disc electrodes 12 mm in diameter. Zn discs were punched from 250 μm-thick foil (Goodfellow), and they were used as anodes in two forms: (i) surface-modified Zn, as described in Section 2.1, and (ii) bare Zn metal, for comparison. Full cells were assembled using Ni/NiOOH cathodes extracted from 2000 mAh Energizer AA commercial batteries through a carefully controlled procedure to preserve their structural and electrochemical integrity. The batteries were first discharged at a C/10 rate to a cutoff potential of 0.15 V to minimize the risk of short-circuiting during subsequent handling and disassembly. Following discharge, the battery casing was removed using a Dremel hand-held precision tool. The cathode material was extracted from the top of the cell, unrolled, and cleaned with deionized water (DI-W). To enhance the mechanical stability and to ensure optima mechanical characteristics, the extracted cathode was subjected to two pressing protocols: cold pressing at 50 kg/cm^2^ psi for 1 min, followed by hot pressing at 80 °C for 5 min at a pressure of 100 kg/cm^2^.

To assess the intrinsic stability and rate capability of the extracted Ni/NiOOH cathodes, half-cell discharge tests were performed in a Pt|Ni/NiOOH configuration at different current densities (Figure S1a). The nearly identical discharge capacities observed confirm that the cathode exhibits stable kinetics and high reversibility. Combined with the excess cathode-to-anode capacity ratio used in the full cells, these results ensure that the cathode does not limit the overall cycling performance. SEM imaging (Figure S1b) further revealed a homogeneous surface morphology of the cathode.

The separators were 260 μm-thick glass microfiber disks (Whatman GF/A) 19 mm in diameter. 80 μL of 4 M KOH aqueous electrolyte was introduced in the cell. The GCPL test was performed with 2 and 4 mAh at 1 C. The cells were galvanostatically charged to the theoretical capacity of Zn (820 mAh g^–1^) and discharged to a cutoff of 1.35 V processes, as reported in a previous literature study.^22^

### 2.3 Electropolymerized Film Characterization

#### 2.3.1 Raman Spectroscopy

Raman spectra of the monomer powder, EPZ electrode, EPZ electrode after CV aging, and CZ and CEPZ electrode cross sections after GCD cycling were recorded by means of a Horiba Jobin Yvon LabRAM microprobe confocal system with excitation at 633 nm provided by a He–Ne laser, delivering 7 mW at the sample surface, with a 600 grid mm^–1^ spectrometer. A 50× long-working distance objective was used.

#### 2.3.2 Atomic Force Microscopy

The surface topography of bare Zn foil, EPZ pristine electrode, and CEPZ after CVs was investigated using a Bruker MultiMode 8 AFM in PeakForce Quantitative Nanomechanical Mapping (QNM) mode under ambient conditions. Images were captured with scan sizes of 10 × 10 μm^2^, a scan rate of 0.6–0.7 Hz, and an image resolution of 512 samples per line. RTESPA-300 antimony (n)-doped Si cantilevers (Bruker, nominal length 125 μm, nominal tip radius 8 nm) were employed. NanoScope Analysis software version 1.5 was used for data processing.

#### 2.3.3 Scanning Electron Microscopy

Cross-sectional imaging of the cycled electrodes was performed to investigate the morphology of the Zn-electrolyte interface after cycling. The electrodes were extracted directly from the disassembled full cells after removal of the Ni/NiOOH cathode, gently rinsed with deionized water, and dried under a vacuum. The Zn anodes were then cut with stainless-steel scissors to expose a clean cross section suitable for observation. The samples were mounted on aluminum stubs by using conductive carbon tape. SEM images were acquired using a field emission-scanning electron microscope (FE- SEM, Zeiss SUPRA 40, Jena, Germany) operating under high vacuum.

## 3 Results and Discussion

### 3.1 Electrochemical Characterizations of the Monomer

Normalized CVs for the VBTMA monomer solution are reported in Figure 1. The CV pattern is characterized by multiple peaks, which can be explained as follows. The first cathodic peak can be associated with the activation of the vinylic terminal of the molecule. Indeed, the first reduction process is related to radical anion formation on the allylic group, an irreversible one-step reaction with the exchange of one electron. The radical anion is stabilized by the phenyl group, owing to a mesomeric effect. The second reduction process is related to the formation of a secondary radical, which can then react with monomers to form the polymeric chain (a possible scheme of the reaction is reported in Figure 2d). Although no anodic peaks were detected, radical cation formation on the allylic group is expected. The absence of any anodic peak in the CV curve suggests that the ammonium group is protected against oxidative attack.^17^

**Figure 1:**
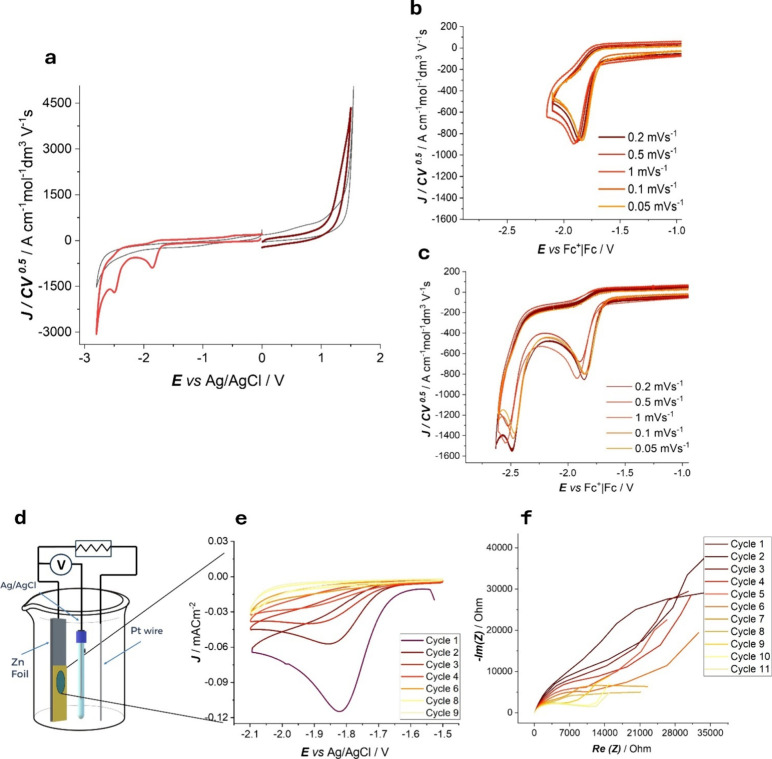
(a) CVs normalized by the scan rate and concentration of the monomer VBTMA in 250 mM LiClO_4_ DMSO at a GC electrode. Scan rate: 0.2 V s^–1^ and (b, c) at different scan rates. (d) Schematic of the electrodeposition setup. (e) Poly­(vinylbenzyl)­trimethylammonium electropolymerization at the GC electrode from 1 mM monomer solution in 250 mM LiClO_4_ DMSO by potentiodynamic cycling at 20 mV s^–1^. (f) Nyquist plots of the EP process from 0 to 12 cycles for VBTMA potentiodynamic electrodeposition in 250 mM LiClO_4_ DMSO on Zn foil. Impendence was recorded after each of the CV cycles reported in Figure S2.

**Figure 2:**
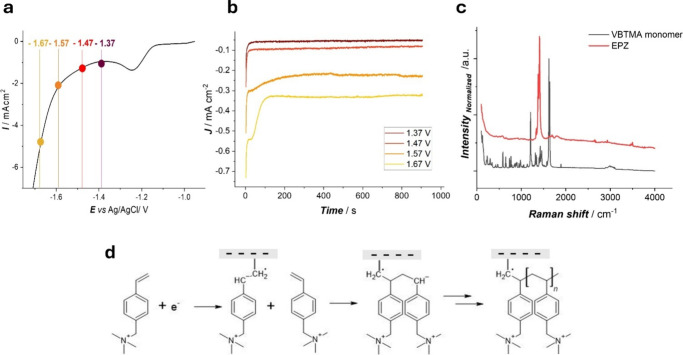
(a) First cathodic-going CV scan for a 0.25 M VBTMA solution in H_2_O:EtOH (9:1 ratio) solvent with 0.05 M NH_4_Cl at a Zn electrode. Scan rate 0.2 V s^–1^, (b) chronoamperometric curves obtained at a Zn electrode with the same electrolyte at different potentials (−1.37, −1.47, −1.57, and −1.67 V vs Ag/AgCl). (c) Raman spectra of VBTMA powder (black plot), EPZ pristine electrode (red plot), and (d) scheme of the electrochemical polymerization reaction occurring at the electrode surface.

### 3.2 Cathodic Electrodeposition of Poly­(vinylbenzyl)­trimethylammonium

#### 3.2.1 Electrodeposition from Organic Solvent-Based Electrolyte

Cathodic electrodeposition of poly­(vinylbenzyl)­trimethylammonium was carried out at different cathodes: GC and Pt, for electroanalytical purposes, and Zn for functional application. Potentiodynamic cycling around the peak associated with activation of the vinylic terminal of the molecule (the first reduction peak, shown in Figure 1b) results in the electrodeposition of the polymer. Typical cyclovoltammograms of the cathodic electropolymerization are shown in Figure 1e. The onset potential for electropolymerization is ca. −1.6 V vs. Ag/AgCl. For VBTMA, under cathodic conditions, polymerization begins through the reduction of the vinylic or allylic double bond, resulting in the formation of a radical anion, as put forward in Section 3.1. Polymerization is then terminated by radical anion recombination or H abstraction. The progressive current decrease found with increasing cycle number is due to polymer formation, which increases ionic resistivity.

This behavior witnesses the formation of an insoluble film giving rise to the growing hindrance of radical formation rate. In order to achieve a better mechanistic understanding, electrodeposition was also performed on Pt and on Zn electrodes (Figure S2) in otherwise identical conditions. It can be noticed that the peak current increases in the order GC > Zn > Pt, denoting the relative catalytic activities of the three cathodic materials. The higher activity of Pt can be straightforwardly related to its ability to adsorb and activate different species, such as hydrogen ions and organic molecules, due to vacant d-orbitals. A faster electrokinetic response is also evidenced by the sharper cathodic peak for Pt. Moreover, the polymerization reaction on Pt is expected to exhibit a shorter termination time. Indeed, according to the above-recalled mechanism, polymerization termination occurs by the recombination of two radical anions or by the H abstraction. When, instead, metallic Zn is employed, a less pronounced cathodic peak is found, in matching with the lower electrocatalytic activity of this metal.

In order to achieve a better mechanistic understanding of the film growth process, we carried out electrochemical impedance spectrometry measured in the same setup after each CV electrodeposition cycle. The Nyquist plots recorded after successive CV cycles (Figure 1f) show a clear trend, consistent with the progressive growth of a polymeric film on the electrode surface.

During the initial cycles (1–2), the impedance spectra exhibit relatively low resistance and a partially developed high-frequency semicircle, suggesting modest charge transfer resistance and minimal mass transport hindrance.

As the number of cycles increases, two main features emerge: a marked increase in total impedance, reflecting the formation of a resistive film, and the development of a low-frequency linear tail with an approximate 45° slope, which can be modeled with a Warburg impedance, indicating the development of diffusion limitations.

These observations suggest that the insulating film increasingly restricts the transport of monomer or supporting electrolyte species to the electrode interface, leading to a transition from a regime with relatively facile kinetics to one dominated by mass transport through the film.

After approximately 10 cycles, the impedance plots tend to stabilize, suggesting the establishment of a self-limiting film structure. Beyond this point, further cycling contributes marginally to the surface coverage, while continuing to increase the overall resistive and capacitive contributions of the film.^16^

A second polymerization protocol relying on potentiostatic conditions was then used for growth onto Zn metal in order to exclude oxidation of the substrate. In this case, a higher concentration of the VBTMA monomer was used (0.25 M instead of 1 mM) in DMSO + 0.05 M of LiClO_4_ and the electropolymerization was done under potentiostatic conditions at −2.1 V vs Ag/AgCl for 30 min. The c.d.-time transient of Figure S3 shows classical nucleation-and-growth behavior, characterized by a c.d. maximum, denoting the attainment of full surface coverage with polymer nuclei, followed by a relaxation to an almost constant value, denoting stable film growth.^19^ Unfortunately, the stability of this type of membrane was found to be really poor. After polymerization, a jelly transparent deposit was observed on the Zn surface, which was then washed out with the solvents. Moreover, yellow coloration of the solution, indicative of carbonium ions and the formation of polymer residues,^15,17^ denotes bulk polymerization in the electrolyte. Comparison of zincate confinement tests for AEI membranes electrodeposited in DMSO- and water-based electrolytes is reported in Figure S5, revealing a higher confinement efficiency for the membrane obtained from the water-based electrolyte.

#### 3.2.2 Electrodeposition from Water-Based Electrolyte

To solve the stability, adhesion, solubility, and bulk polymerization issues of membranes formed in the DMSO-based electrolyte, a modified electrodeposition strategy was developed, using a water-based electrolyte.

Specifically, we implemented a procedure inspired by refs 19,20 . In this case, the polymerization solution contained 0.25 M VBTMA monomer. The solvent was a 9:1 ratio volume of DI water and ethanol, and the supporting electrolyte was 50 mM ammonium chloride.

In Figure 2a, we report the cathodic-going scan of an LSV measurement carried out with this system. It is worth noting that the cathodic behavior of VBTMA in water-based solution shows some differences with respect to that observed in DMSO. In fact, in the former case, only one reduction peak is found before the quasi-Tafel HER c.d. increase. Since DMSO is an aprotic solvent, it can stabilize the radical anion formed during the first electrochemical reduction process.

Instead, in the aqueous electrolyte, the reaction of the radical anion with protons is predominant. For this reason, the formation of the radical anion cannot be detected with a voltammetric scan. According to ref 19, the optimal electrochemical conditions for the deposition were achieved through potentiostatic deposition at −1.47 V vs Ag/AgCl, at a temperature of 50 °C for 15 min with stirring. In our investigation, we extended the testing range in view of gaining more insight into the film growth process: The potentials highlighted with colored dots were chosen for the potentiostatic electrodeposition tests. The electropolymerization potentials (1.47 and 1.67 V vs Ag/AgCl)—both within the HER range—were chosen to assess the effect of hydrogen evolution rate on the formation and stability of the anionic membrane.

Figure 2b depicts the chronoamperograms measured as the abovementioned potentials. At variance with the behavior observed in the DMSO solution (Figure 3), a sharp decrease in c.d. is found after a few minutes of electodeposition. Eventually, the current density exhibits a slowly decreasing value—in dependence of the applied potential—indicating stable growth of a polymer with a progressively increasing resistance, though in the presence of concurrent HER. The film formed at −1.67 V showed less coverage than the one at −1.57 V probably because at higher cathodic potential, a side reaction like HER occurs. We selected potentiostatic deposition at −1.47 V vs Ag/AgCl for systematic electrochemical tests because this potential balances the competing effects of polymer growth rate and parasitic HER, prompting uniform membrane coverage.

**Figure 3:**
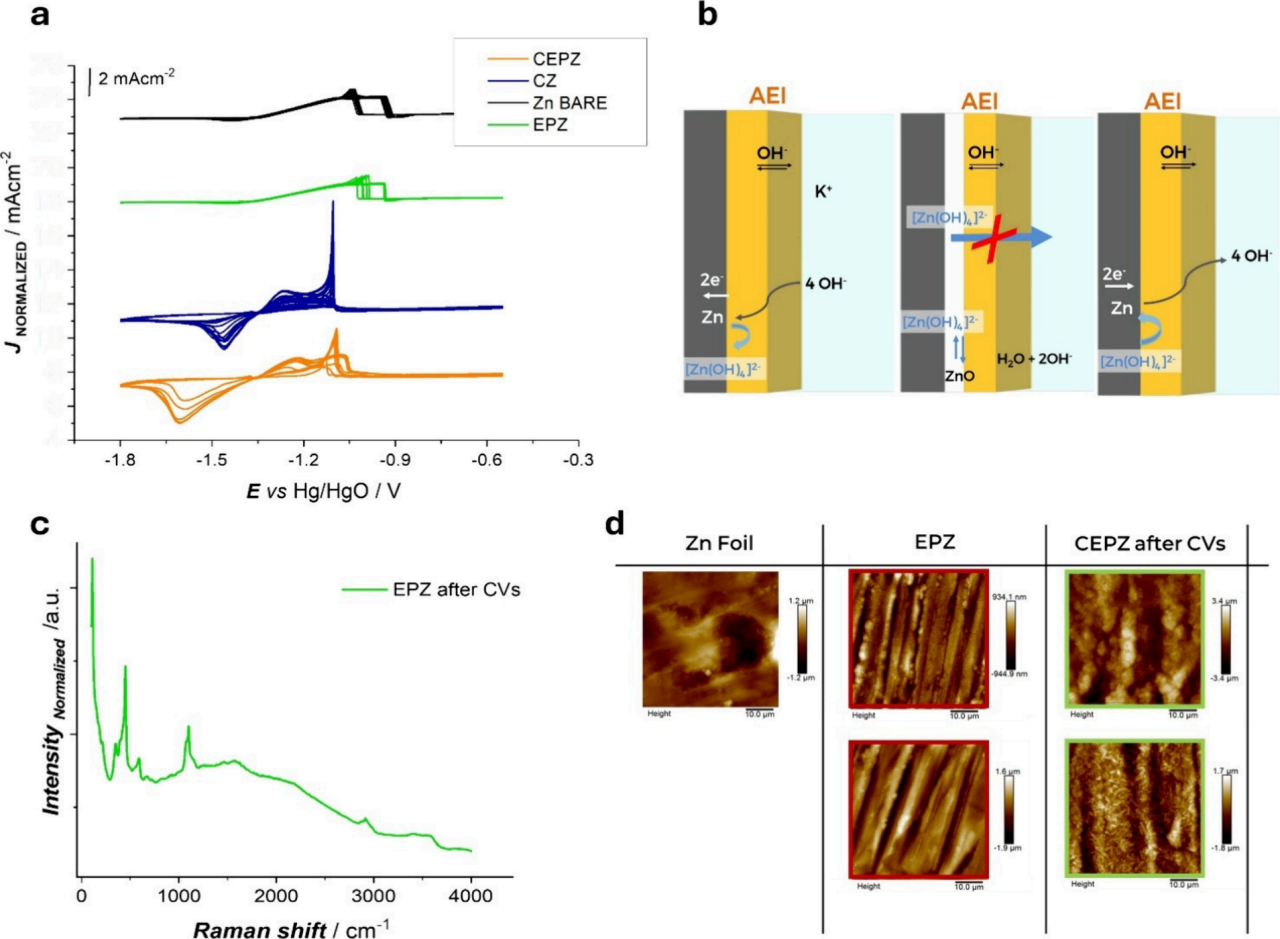
(a) Corrosion resistance cyclovoltammetric tests, performed on bare and surface-modified Zn foil in 4 M KOH (scan rate of 4 mV/s): bare Zn foil (black line), EPZ in H_2_O:EtOH (green line), CZ (blue line), and CEPZ (orange line). (b) Schematic illustration of the zincate confinement effect. (c) Raman spectra of the EPZ electrode after CV cycling (orange plot). (d) AFM images of bare Zn foil, EPZ pristine electrode, and CEPZ after CVs cycling.

Electrodeposition can follow a different growth mechanism. Indeed, Sekine et al. reported that the organic layer formed during polymerization occurs via an ionically conducting layer at the surface.^20^ The organic layer is not insulating, but ionically conducting.

### 3.3 Characterization of Electrodeposited Poly­(vinylbenzyl)­trimethylammonium Films on Zn

3.3

#### 3.3.1 Raman Spectroscopy

Figure 2c reports the Raman spectra of the VBTMA monomer and electropolymerized poly­(vinylbenzyl)­trimethylammonium. The spectrum of the monomer powder is dominated by the aromatic bands (ring bend vibrations: 470 and 587 cm^–1^; C–H wagging: 733, 772, and 880 cm^–1^; C–H bending and C–C aromatic vibration: 1315 and 1341 cm^–1^; benzene ring stretching: 1402 cm^–1^; C=C stretching: 1619 cm^–1^), along with contributions from the olefinic groups (C–H_2_ wagging: 901 and 1880 cm^–1^; C–H stretching: 3000 and 3070–3100 cm^–1^; C=C stretching: 1638 cm^–1^). The spectrum also shows C–N symmetric stretching bands (650, 847, and 948 cm^–1^), aliphatic bands related to C–H_2_ deformations (1427 cm^–1^), and quaternary ammonium bands related to C–N symmetric stretching (650 cm^–1^) and NR_4_ deformation (1467 cm^–1^). The list and attribution of the monomer bands are reported in Table 1. This peak assignment is based on the molecular structure of the molecule and on reported literature.^23^

**Table 1:** List of VBTMA Raman Bands and Their Attribution to Vibrational Modes

Raman shifts (cm^–1^)	assignment
470	ring bending (aromatic)
587	ring bending (aromatic)
650	C–N symmetric stretching
733	C–H wagging (aromatic)
772	C–H wagging (aromatic)
847	C–N symmetric stretching
880	C–H wagging (aromatic)
901	C–H_2_ wagging (olefin)
948	C–N symmetric stretching
980	C–H wagging (olefin)
1207	terminal olefin or aryl C–N stretching
1300–1400	C–H bending + C–C aromatic vibration
1423	C–H_2_ deformation (olefin)
1467	NR_4_ deformation
1619	C=C stretching (aromatic)
1638	C=C stretching (olefin)
1880	C–H_2_ wagging (olefin)
2850–3000	C–H stretching
3070–3100	C–H_2_ asymmetric stretching (olefin)

Upon electrochemical polymerization of VBTMA, the Raman spectra of the resulting polymer membrane show significant changes compared to the monomer. In the monomer spectrum, some peaks associated with the vibrational modes of the vinyl group, the benzyl ring, and the trimethylammonium moiety are observed. However, in the polymer spectrum, only three dominant peaks remain, located at 1332, 1380, and 1409 cm^–1^, while the other peaks present in the monomer spectrum disappear. This disappearance of peaks in the polymer can be attributed primarily to chemical changes brought about by the polymerization process, structural reorganization, and orientation effects during polymerization and deposition onto the metal electrode.

The peaks at 1334, 1377, and 1407 cm^–1^ likely correspond to vibrational modes observed in the monomer spectrum but appear red-shifted in the polymer backbone. (i) The 1334 cm^–1^ peak can be associated with C–C stretching within the polymer backbone, especially in conjugated or extended structures that form during polymerization. (ii) The 1377 cm^–1^ peak corresponds to CH_2_ scissoring or deformation in the polymer chain, indicating the preserved presence of the alkyl components of the trimethylammonium group. (iii) The 1407 cm^–1^ peak could be linked to C–H bending or ring deformation from the benzyl group in the polymer, as it retains its rigidity and contributes to the vibrational activity in the polymer backbone.

The disappearance of most monomer peaks is likely due to surface selection rules since the polymer chains are expected to adsorb onto the electrode surface and thus to take on a parallel orientation without optically active vertical components. Additionally, the polymerization process itself leads to changes in the vibrational freedom of the molecule, as the vinyl group of the monomer is consumed during polymerization, resulting in fewer active vibrational modes in the polymer spectrum.^23,24^

#### 3.3.2 Zincate Confinement Tests

Electrochemical measurements addressing the evaluation of zincate confinement were conducted using a three-electrode electroanalytical setup with Hg/HgO as the reference electrode.

Electropolymerized quaternary ammonium AEI coatings were deposited on Zn rod and Zn foil electrodes to serve as confinement membranes for [Zn­(OH)_4_]^2–^ ions as described in Section 2.1 and in the scheme reported in Figure[Fig fig3]b.

Experiments with Zn rods were conducted in the initial phase of our research to provide a rapid assessment of the reliability of the confinement effect under controlled electrochemical conditions. For comparison, a commercially available AEI-type quaternary ammonium coating (PiperION) was applied via dip-coating (see Section 2.1) to evaluate differences in performance between the electropolymerized film and the commercial membrane.

To assess the impact of AEI coatings on Zn dissolution and deposition during charge and discharge cycles, cyclic voltammetry experiments were performed with a host stagnant and stirred electrolyte: Of course, the latter condition is meant to ease zincate ion transport from the electrode surface to the bulk solution.

Figure S4 presents a selection of representative CV curves (see Section 2.2.3) for various Zn electrodes: uncoated (black curve), **EPZ** (pink curve), **DZ** (red curve), and **DEPZ** (green curve). The scans start from the open circuit potential (OCP) value of −1.39 ± 0.03 V vs Hg/HgO and then progress toward more positive potentials. For the bare Zn electrode, an increase in current density during the anodic sweep between −1.35 and −1.07 V corresponds to Zn oxidation, releasing zincate species into the alkaline electrolyte. A sharp drop in current density at ∼−1.02 V marks the formation of a partially passivating ZnO layer, accompanied by characteristic periodic current relaxation oscillations. The passive film (ZnO/Zn­(OH)_2_) typically forms, breaks down, and reforms repeatedly, leading to periodic spikes and drops in the current. These oscillations are commonly associated with autocatalytic processes, where local dissolution of the passive layer is followed by fresh metal exposure, triggering reoxidation (e.g., ref 25 and references therein).

The **DZ** electrode shows a pronounced oxidation peak at −1.19 V, followed by a region of high current density with a declining I–V slope, characteristic of active corrosion and accumulation of corrosion products.^9^ In contrast, **EPZ** electrodes demonstrated poor mechanical stability; the AEI film delaminated due to the abrupt volume changes associated with ZnO formation.

To address this, **DEPZ** electrodes were tested, using the commercial polymer to enhance mechanical strength. This modification prevented film peeling and maintained the confinement function during cycling. On the cathodic sweep, **DEPZ** exhibited a sharp deposition peak between −1.35 and −1.45 V, with a current density five times higher than the uncoated electrode, where the cathodic peak was barely visible. **DEPZ** also showed stable anodic and cathodic peak currents during cycling, suggesting an enhanced Zn deposition efficiency and confinement of soluble zincate ions near the electrode.

Following this coating screening, systematic tests were conducted using Zn foil electrodes and a Celgard membrane for mechanical stability. The porous Celgard layer replaced the anionically conductive PiperION to decouple the mechanical reinforcement from the ionic conductivity effects. This allowed the evaluation of the electropolymerized AEI coating confinement properties under practically relevant operating conditions while minimizing mechanical degradation. Cyclic voltammograms for bare Zn foil (black curve), **EPZ** (red curve), **CZ** (blue curve), and **CEPZ** are shown in Figure 3a. The bare Zn foil and **EPZ** exhibited anodic and cathodic behaviors similar to those of their Zn rod counterparts. **CZ** displayed lower anodic currents due to the lower ionic conductivity of the Celgard layer, with both anodic and cathodic peak currents decreasing over cycles and eventually vanishing. In contrast, **CEPZ** demonstrated two distinct anodic peaks, reflecting active corrosion and subsequent precipitation. Notably, **CEPZ** exhibited stable anodic and cathodic peak currents throughout cycling.

In summary, the **DEPZ** and **CEPZ** electrodes showed superior cycling stability. Bare Zn electrodes suffered free dissolution, as [Zn­(OH)_4_]^2–^ ions flowed freely to the bulk solution. Anionic membranes, by contrast, slowed marked beneficial zincate confinement to the electrode surface, resulting in notably enhanced cycling performance. The DEPZ and DZ configurations were part of preliminary screening tests (Figure S4) aimed at assessing zincate confinement and confirming the validity of our electrochemical approach. Since comparable results and mechanical stability were later achieved using the Celgard-supported configuration, the CEPZ design was ultimately selected for in-depth investigation due to its lower material cost, easier processing, and higher scalability for practical electrode fabrication. Confinement tests with the commercial AEI served as a preliminary screening reference. This assessment enabled full-cell cycling tests in which only the electropolymerized membrane was used.

#### 3.3.3 GCD Cycling

Alkaline Ni–Zn full cells were assembled using a split-cell setup with a Ni­(OH)_2_ cathode. This design was specifically chosen to restrict the electrolyte volume, thereby simulating conditions closer to practical applications. This results in testing conditions that are notably harsher than those customarily adopted in the literature.

It is acknowledged that employing a cathode extracted from a commercial Ni–MH battery may pose a limitation in terms of interlaboratory reproducibility. However, control experiments (Figure S1) confirmed its electrochemical stability and reproducibility, validating its use as a reliable platform for the comparative anode study presented in this work.

The cells were charged at a constant current to 2 mAh and discharged to 1.35 V at a 1 C rate with the upper voltage cutoff set at 2.1 V to prevent degradation of the electrolyte.

Figure 4a,b presents the galvanostatic charge–discharge profiles for cells utilizing CEPZ and CZ electrodes. To provide a baseline for comparison, a reference cell containing bulk Zn foil was tested under identical conditions. The Zn foil electrode (black line in Figure 4b) maintained stable cycling for only 14 cycles before experiencing a rapid decline in the capacity. This behavior can be attributed to the formation of a dense ZnO passive layer, which inhibits ion transport and leads to premature capacity loss.

**Figure 4:**
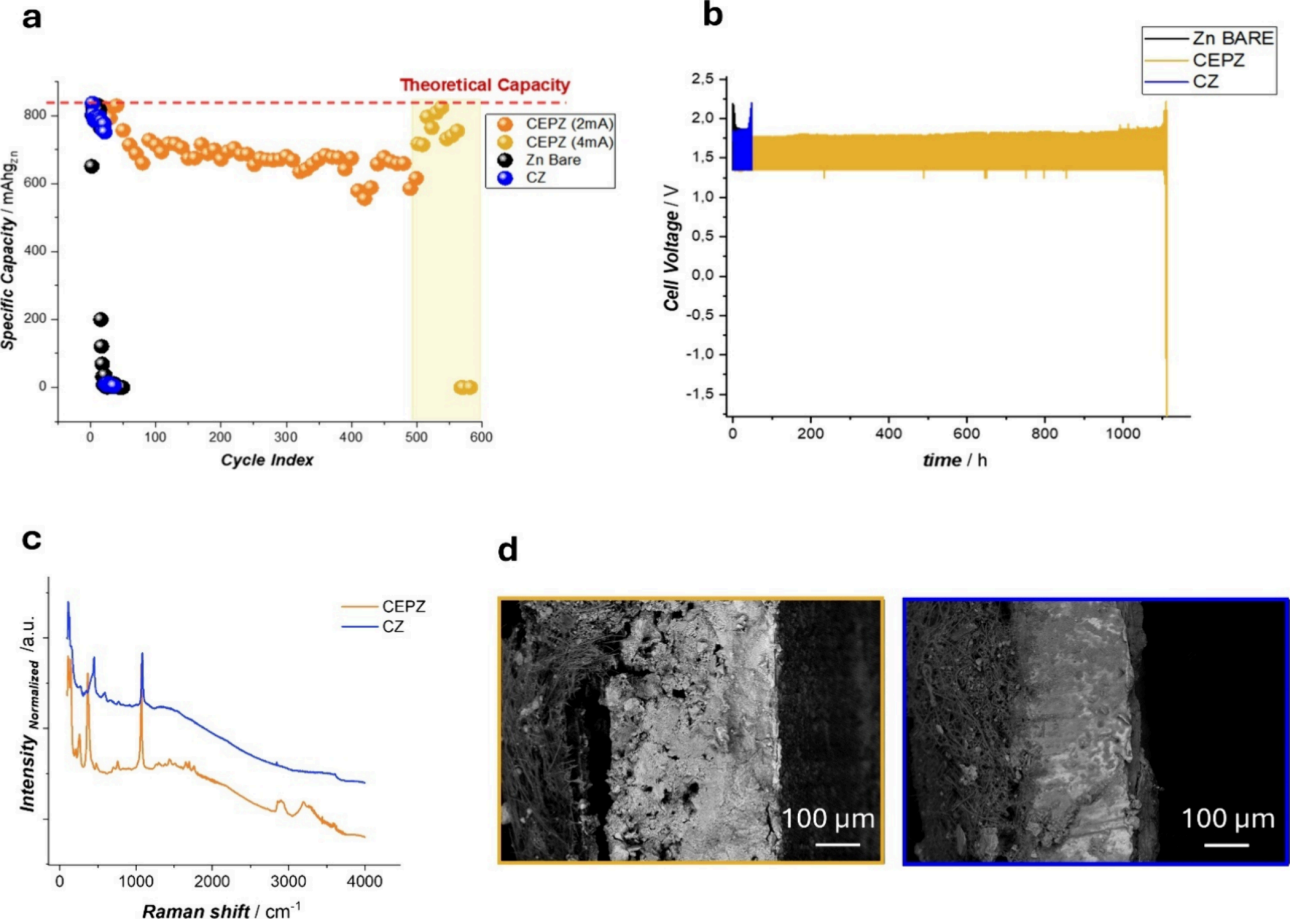
Specific discharge capacity vs cycle index (a) and potential-time time-series (b) of Zn–Ni full cells with bare Zn, CZ, and CEPZ anodes. (c) Raman spectra of CZ and CEPZ electrodes after GCD cycling. (d) Cross-sectional SEM of Zn anodes after cycling: (left) CEPZ (extended cycling), (right) CZ (early passivation). The filamentous object is the GF separator.

In comparison, the CZ electrode delivered enhanced performance, sustaining 30 cycles before significant capacity fading occurred. This improvement highlights the ability of the Celgard layer to exert a moderate confinement effect, restricting the dissolution and flux of [Zn­(OH)_4_]^2–^ ions to the bulk and thereby contributing to improved Zn anode stability.

Notably, the introduction of the CEPZ electrode yielded a dramatic enhancement of the cycling stability. The CEPZ-based cells demonstrated a prolonged cycle life exceeding 500 cycles: approximately 20 times longer than that of the CZ electrode. This significant improvement aligns with the cyclic voltammetry data in Figure 4b and underscores the superior confinement provided by the electropolymerized membrane. By effectively localizing zincate ions near the electrode surface and reducing their loss to the bulk electrolyte, the CEPZ membrane minimizes Zn dissolution, delays passive layer formation, and ensures a stable anode performance over extended cycling. The same CEPZ electrode was even cycled with higher current achieving 4 mAh from cycle 500 (highlighted yellow square Figure 4a), demonstrating even in this case good cycling stability for about 100 cycles. The Coulombic efficiency of the CEPZ electrode is reported in Figure S6, showing a capacity efficiency of about 86% over more than 500 cycles.

Cross-sectional SEM (Figure 4d) shows the morphological counterparts of the electrochemical trends. In the uncoated Zn anode, which exhibited a rapid capacity fade, a continuous and compact layer is observed at the metal side of the separator. This layer is consistent with the formation of a dense ZnO/Zn­(OH)_2_ layer acting as a transport-blocking passivation film. In contrast, the electrode bearing the electropolymerized coating, which sustained long-term cycling, displays a more graded and less compact interphase at the Zn side, consistent with the higher Zn utilization and extended cycling stability observed electrochemically.

#### 3.3.4 Long-Term Stability of EPZ Electrodes

Benzyltrimethylammonium-type anion-exchange polymers are the object of active research for alkaline membrane fuel cells and water electrolyzers.^26^ However, they suffer from degradation under alkaline conditions. For this reason, to evaluate the impact of cycling in alkaline electrolyte, we have performed Raman spectroscopy of EPZ electrodes after multiple CVs reported in Section 3.3.2 (Figure 3c). The Raman spectra of the aged electrodes are dominated by ZnO bands in the range corresponding to the Zn–O bending and stretching modes (∼350–600 cm^–1^). The strong peak observed at 455 cm^–1^ can be assigned to the E2 optical mode of ZnO, while the smaller peak at 356 cm^–1^ can be assigned to the E2 High–E2 Low multiphonon process. Notably, the region before the peak at 455 cm^–1^ shows a broadened structure due to spectral overlap of multiple peaks related to the AEI membrane.

Additionally, the peak at 587 cm^–1^ can be assigned as the E1­(LO) mode, denoting impurities and structural defects. These can derive from doping resulting from the electrochemical oxidation of Zn in the presence of the AEI membrane such as oxygen vacancies.^9^

Peaks at 1070 and 1100 cm^–1^ are related to multiphonon mode (E_2H_–E_2L_) in C-doped ZnO.

In addition, polymer-related bands can also be measured after aging. Peaks at 2885 and 2909 cm^–1^ represent C–H stretching from the quaternary ammonium methyl groups and are present at the same position in the monomer spectra. Additional O–H stretching modes were detected at 3496 and 3580 cm^–1^, suggesting the presence of Zn­(OH)_2_ complexation, as well as free or weakly bonded OH^–^ ions.

It is important to note that the Raman spectra of the EPZ electrode, reported in Section 3.3.1, show only three peaks located at 1334, 1377, and 1407 cm^–1^. In contrast, upon aging and the formation of ZnO, additional Raman peaks associated with the polymer become evident that were not detectable in the pristine sample. This can be explained by the fact that, upon formation of a ZnO layer, polymer chains are displaced from the metal surface, taking on a bulk rather than an adsorbed chain arrangement. As a result of this, surface selection rules no longer apply, and the full Raman spectrum can be measured.

To investigate the surface morphology and assess the retention and morphology of the electrodeposited polymer after electrochemical cycling, AFM images were obtained for the EPZ and CEPZ samples as well as for a bare Zn foil as a reference. The AFM images of the EPZ Figure 3d (blue rectangle) sample exhibit well-defined, grooved textures across the surface. These structures that cannot be found at the surface of the pristine Zn foil are likely associated with bubble paths sweeping across the electrode, resulting from HER concurrent with polymer electrodeposition in the aqueous electrolyte. After electrochemical cycling, the AFM images of the CEPZ sample still display similar ridged features, indicating that the polymer is still present at the electrode surface after plating–stripping cycles. However, the formation of finer granular structures on top of the original fibrous structure indicates some degree of morphological rearrangement of the polymer, brought about by cycling.

These morphological changes are compatible with the modifications in the Raman spectral pattern of Figure 3c. AFM analysis provided evidence of the homogeneity and continuity of the electropolymerized films.

To further investigate the interfacial chemistry after prolonged galvanostatic cycling, Raman spectra were acquired for the bare CZ and CEPZ anodes extracted from the aged cells, as reported in Figure 4c.

Both samples display strong features in the 350–600 cm^–1^ region, corresponding to Zn–O lattice vibrations. These can be assigned to the E_2_ (high) mode of ZnO at ≈455 cm^–1^ and the E_1_(LO) mode at ≈570–590 cm^–1^, typical of defective or doped ZnO formed under electrochemical conditions, coherently with the results obtained with CEPZ after CVs (Figure 3c). The spectrum of the bare Zn electrode (CZ) is dominated by these ZnO-related peaks with a sharp and intense E_2_ band, consistent with the formation of a dense and crystalline ZnO passivation layer.

In contrast, the polymer-coated electrode (CEPZ) shows broader ZnO-related bands and several additional peaks between 1260 and 1760 cm^–1^, which can originate from the polymeric film and partially oxidized organic species. Specifically, the bands at 1262 and 1300 cm^–1^ correspond to C–N and C–C stretching within the polymer backbone and those at 1385 and 1447 cm^–1^ are associated with CH_2_ scissoring and NR_4_^+^ deformation modes. The peaks at 1514, 1655, and 1695 cm^–1^ can originate from the C=C stretching of the aromatic ring and the C=O vibrations of oxidized fragments formed during cycling. Additional features at 1762 cm^–1^ (C=O stretch) and in the 2850–2930 cm^–1^ region (C–H stretching from methyl groups) confirm the persistence of the organic component. Broad O–H stretching bands at ≈3195–3300 cm^–1^ indicate the presence of Zn­(OH)_2_ and adsorbed water within the interphase.

The coexistence of polymer- and ZnO-related bands in the Raman spectrum of CEPZ witnesses that the electropolymerized membrane is still present after extensive cycling, forming a mixed organic–inorganic interphase. This hybrid layer likely consists of a thin ZnO/Zn­(OH)_2_ network embedded or overlaid by the polymer film, which confines corrosion products and mitigates the formation of a dense passivation layer. These findings are consistent with cross-sectional SEM observations reported in Figure 4d, where the coated anode shows a graded and less compact interphase compared with the fully passivated structure of the bare Zn electrode.

## 4 Conclusions

In view of enhancing the cycling stability of Zn anodes in alkaline batteries by surface modification of the metal, in this study, we investigated the electrochemical behavior and electrodeposition of poly­(vinylbenzyl)­trimethylammonium on various cathodic materials, including glassy carbon (GC), platinum (Pt), and zinc (Zn).

The film growth process was studied through cyclic voltammetry and electrochemical impedance spectroscopy, allowing the identification of optimal electropolymerization conditions.

Moreover, organic and water-based electrodeposition bath chemistries were considered to achieve high mechanical properties of the membranes.

The best electropolymerization conditions resulted in −1.47 V vs Ag/AgCl in water–ethanol electrolytes.

In addition, we employed Raman spectroscopy to qualify the VBTMA electropolymerization process.

After optimizing the electropolymerization process at Zn cathodes, we investigated the use of poly­(VBTMA) films as anion-exchange membranes on Zn electrodes, assessing their ability to confine zincate ions. The results showed that electropolymerized membranes significantly improved the cycling stability of Zn-based anodes in the free electrolyte. Functional performance could be further enhanced by wrapping in a Celgard film.the Zn anode bearing the electropolymerized coatings.

Finally, cycling tests of Zn–Ni full batteries revealed a significant enhancement (of a factor of more than 35) in the long-term stability of electrodes with electropolymerized VBTMA coatings. This performance underscores the potential of poly­(VBTMA)-based coatings as effective anion-exchange membranes for zinc anodes in energy storage systems

These findings demonstrate the potential of electropolymerized coatings for enhancing the performance and longevity of zinc-based electrochemical energy storage devices.

## Supplementary Material



## Notes

Additional experimental procedures, electrochemical data, and complementary characterization results; representative electrochemical measurements; supplementary figures; and discussion of Zn anode performance (PDF)
