# Supplementary material for: Improving Zn Anode Cyclability in Alkaline Electrolytes with Electropolymerized Anion-Selective Film
Source: ACS Appl Energy Mater. 2025 Nov 18;8(24):17659–69. doi: 10.1021/acsaem.5c02209 (PMC12728797; doi:10.1021/acsaem.5c02209)
Supplement: Supplementary file 1 [file ae5c02209_si_001.pdf]

Supporting Information:

Improving Zn anode cyclability in alkaline electrolytes with electropolymerized anion-selective film.

Elisa Emanuele[1*], Claudio Mele[2], and Benedetto Bozzini[1]

[1]Department of Energy, Politecnico di Milano, via Lambuschini 4, 20156 Milano, Italy
[2]Department of Innovation Engineering, University of Salento, Via Monteroni, 73100 Lecce, Italy

* Corresponding author: elisa.emanuele@polimi.it, ORCID ID: 0000-0002-0241-8924.

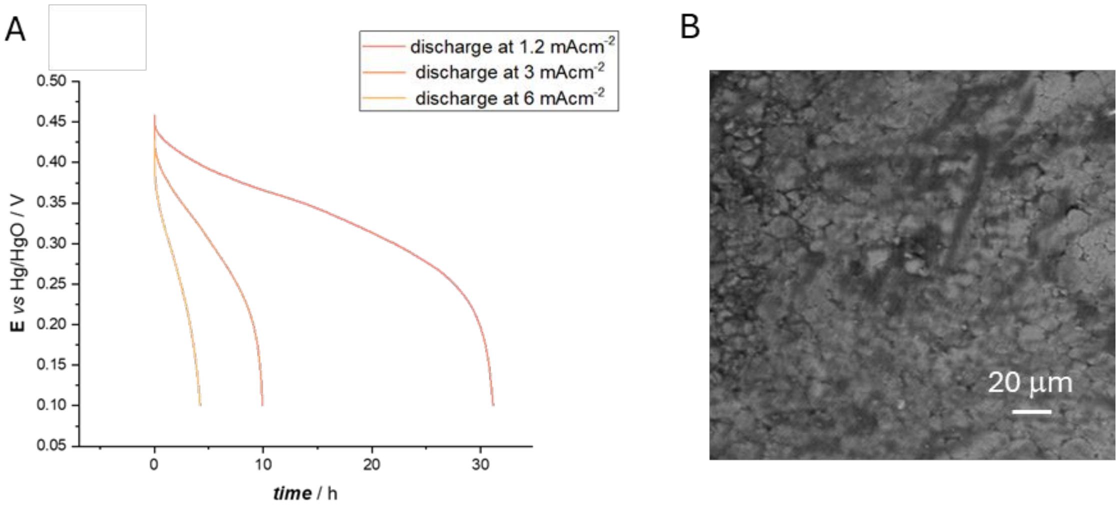

Figure S1 – Characterization of Ni/NiOOH cathode extracted from commercial AA Ni–MH batteries. (a) Galvanostatic discharge curves, measured at different current densities (1.2, 3, and 6 mA cm^{-2}), 100% DOD in 6 M KOH electrolyte. (b) SEM micrograph of as-extracted cathode.

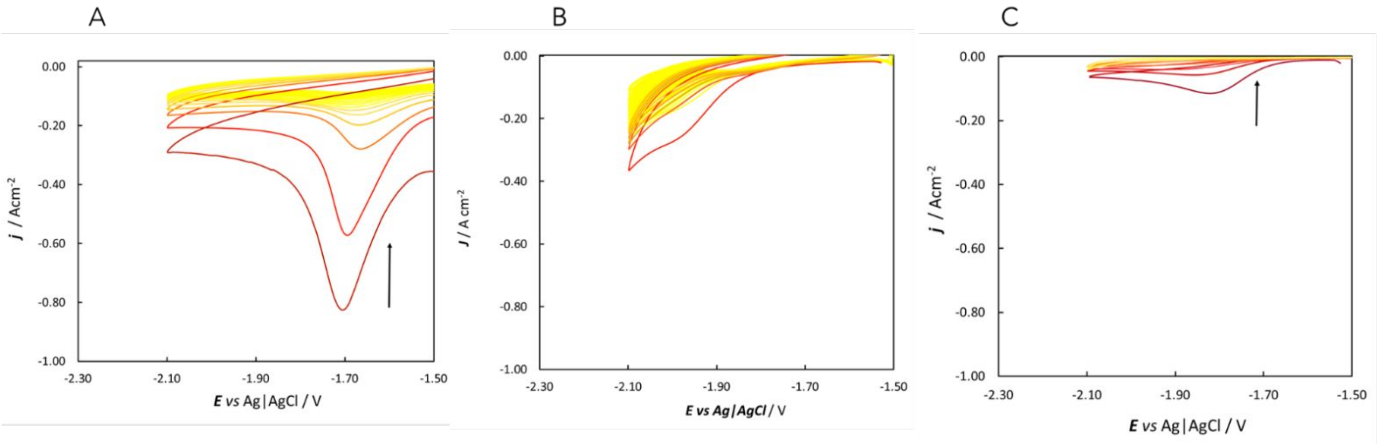

Figure S2 – Poly-(vinylbenzyl)trimethylammonium electropolymerization at Pt (A) and Zn (B) electrode. Data for the GC electrode, replotted from Figure 3, are provided in Panel (C), for comparison.

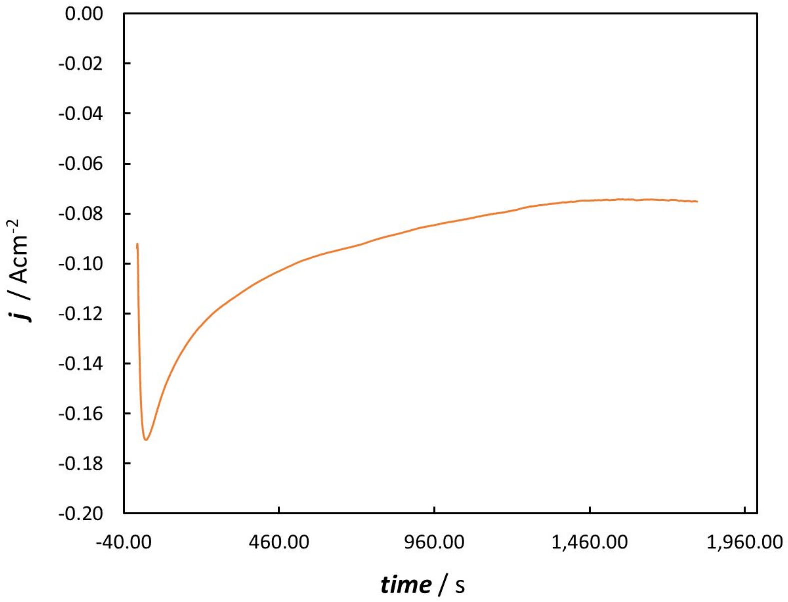

Figure S3– Chronoamperogram for the electrodeposition of Poly-(vinylbenzyl)trimethylammonium at -2.1V *vs* Ag/AgCl at Zn from 0.25 M VBTMA in 0.05M $LiClO_4$ DMSO at 25°C.

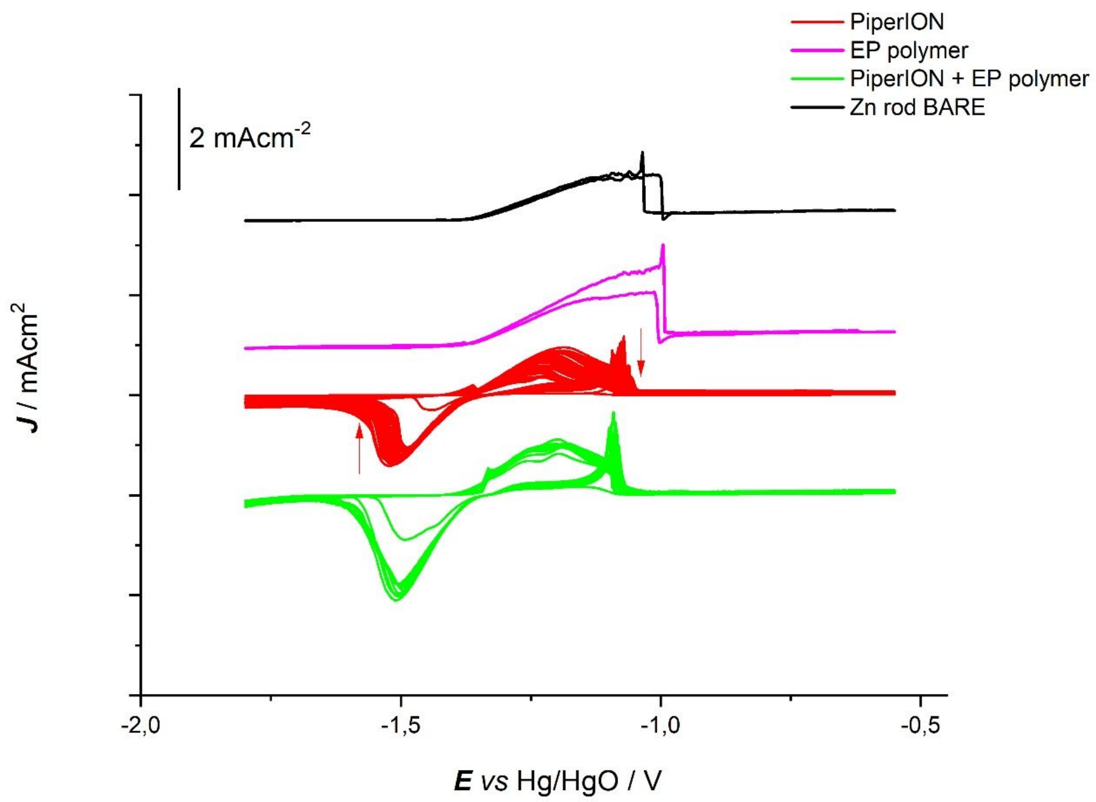

Figure S4 –Corrosion resistance tests performed on Zn rod as working electrode in 4M KOH, multiple cycling at 4 mV/s potential scan rate. Zn rod bare (**black** line), Zn with EP in DMSO (**pink** line), Zn with Emanuele's polymer (**red** line), Zn with EP in H_2O:EtOH and Emanuele's polymer (**green** line).

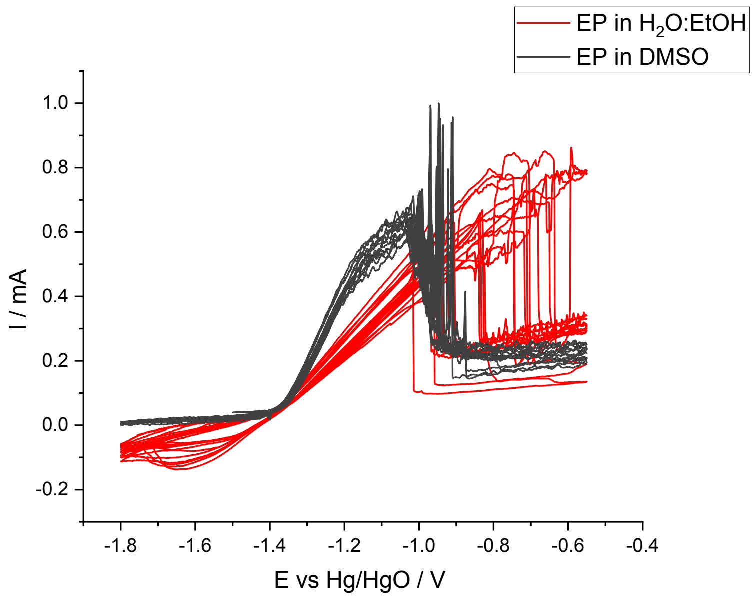

Figure S5–Comparison of corrosion resistance tests performed on a Zn rod working electrode in 4M KOH, multiple cycling at 4 mV/s scan rate. Zn rod with electropolymerized membrane in DMSO electrolyte (**black** line), and in water based electrolyte (red line).

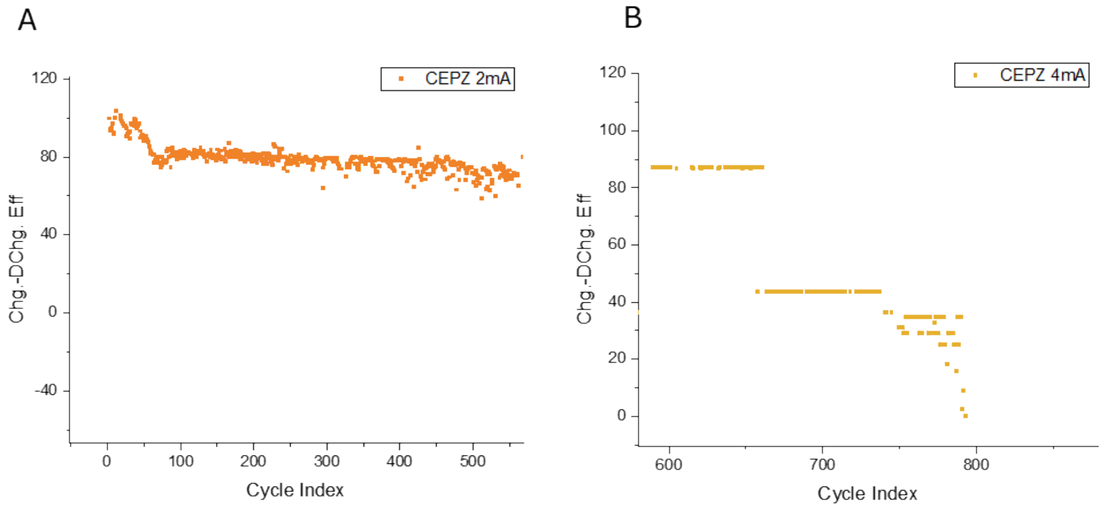

Figure S6– Coulombic efficiency of CEPZ electrode full-cell as a function of the cycle number, at 2 mA (A) and at 4mA (B).
